# Lipoprotein(a): an independent risk factor for ischemic heart disease that is dependent on triglycerides in subjects with type 2 diabetes mellitus

**DOI:** 10.1186/1476-511X-6-26

**Published:** 2007-10-02

**Authors:** Ali AlBahrani, Mohammed Alkindi, Eileen Marks, Said AlYahyaee, Alan Shenkin

**Affiliations:** 1 Department of Chemical Pathology, St Mary’s Hospital, Newport PO30 5TG, Isle of Wight, UK; 2 Department of Clinical Biochemistry, Sultan Qaboos University Hospital, Muscat, Sultanate of Oman; 3 Department of Biochemistry, Royal Liverpool University Hospital1, Duncan Building, 4^th^ floor, L69 3GA, Liverpool, UK

## Abstract

Lipoprotein(a) is an independent risk factor for Ischaemic Heart Disease (IHD) in the general population. There are conflicting reports in the extent of its association with IHD among subjects with Type 2 diabetes mellitus (T2DM).

The aim was to determine the concentration of Lp(a) and its relationship with other lipids parameters among Omani T2DM subjects with and without IHD. An over-night fasting blood sample from 221 T2DM subjects (86 females and 135 males) and 156 non-diabetics (69 females and 87 males) aged 30–70 years (as control) was taken for lipid profile studies.

Lp(a) was significantly lower (p = 0.012) among T2DM subjects 0.123(1.12) g/L compared to non-diabetics 0.246 (1.18)g/L, irrespective of gender.

A significant correlation (Spearman correlation, P = 0.047) was revealed between Lp(a) and IHD among Omani T2DM subjects. The proportions of T2DM subjects with IHD and an Lp(a) >0.3 g/L was higher compared to T2DM without IHD irrespective of gender, for women 42% vs. 27% and for men 17.5 vs. 8%, respectively.

A significant negative correlation existed between Lp(a) and triglycerides (r = 0.41, P = 0.002) among T2DM subjects. In contrast, a significant positive correlation existed between Lp(a) and LDL-chol among the non-diabetic subjects.

Women had significantly higher Lp(a) concentration compared to men ( 0.30 Vs. 0.16 g/L, P < 0.0001) irrespective of the diabetic status.

Lp(a) is an independent risk factor for IHD among Omani T2DM subjects. Lp(a) concentration was significantly lower and negatively correlated with triglycerides among Omani diabetic compared to non-diabetic subjects.

## Introduction

Lipoprotein(a) is an LDL-like particle in which apo(a)- a glycoprotein consisting of several repetitive kringle structures – is attached to apolipoprotein B by a disulphide linkage [1]. Elevated concentration of Lp (a) has been identified by a meta-analysis as a modest risk factor for IHD in the general population [2,3]. There are contradictory reports in the literature in terms of Lp(a) concentration among T2DM subjects, some studies reported lower [3-7], others reporting higher, concentration compared to non-diabetic subjects [8-10]. In terms of its relationship with IHD, again controversy exists in the literature [11-13].

Variations in Lp(a) levels are mainly genetically determined [14]. Other factors like the diet, drugs, hormones and glycemic control were also found to effect Lp(a) concentration [15,16]. Lp(a) has also been found to be influenced by other lipid parameters an inverse relation was revealed between Lp(a) and triglycerides and positive correlation with total and LDL cholesterol among diabetic subjects [17]. Therefore, this study was undertaken to investigate the level of Lp(a) as well as its relationship with other lipid parameters in a cohort of type 2 diabetic subjects with and without IHD.

## Methods

The institutional Ethics Committee approved the study and all subjects gave their informed consent prior to participating in this study. Subjects (221 diabetics, 86 women and 135 men) aged 30–70 years attending Lipids and Diabetes clinics at Sultan Qaboos University Hospital were selected for the study. 156 healthy (non-diabetics), age matched, randomly selected volunteers from the thyroid clinic (euthyroid subjects and under regular follow-up), within the department and blood donors were selected as control. Diagnosis of T2DM was based on clinical characteristics, prior history of use of oral hypoglycaemic agents, the presence of obesity, no history of ketosis, or strong family history of diabetes. World Health Organization criteria for diagnosis of T2DM either by an abnormal oral glucose tolerance test (OGTT), two abnormal fasting blood glucose (>7.0 mmol/L) or high HbA1_c _(>7%) were fulfilled by all patients with type 2 diabetes. The proportions of T2DM subjects on insulin, oral hypoglycaemic agents or dietary control were 3%, 57% and 40%, respectively. History of smoking, alcohol, exercise and prophylactic drugs (e.g. β-blockers, aspirin, diuretics and sulphonylureas) was collected. Blood pressure was measured using a digital sphygmomanometer with the patient in the sitting position. Diagnosis of IHD was based on having a myocardial infarction three months prior entry to the study, stable angina pectoris with positive thallium stress test, coronary angiogram and previous history of Percutaneous Transluminal Coronary Angioplasty (PTCA). Exclusion criteria were myocardial infarction within three months prior to entry to the study, uncontrolled thyroid disease (hypo or hyperthyroidism), proteinuria (positive urine protein dip-stick ×2), severe hepatic impairment (chronic active liver disease or those individuals with obstructive liver pattern), chronic kidney disease (CKD) (creatinine level >114 μmol/L) and lipid lowering agents.

Cholesterol (c), triglyceride (TG), and glucose measurements were performed using timed endpoint enzymatic methods on the Synchron CX system (Beckman, Richmond, California). Within and between batch precisions for cholesterol (4.3 mmol/L) were 3% and 4.5%, respectively, for Tg (2.0 mmol/L) were 3% and 4%, respectively; and for glucose (5.5 mmol/L) were 2% and 3%, respectively.

HbA1_c _concentration was determined as a percentage of total haemoglobin in whole blood on the Synchron CX system (Beckman, Richmond, California) with within and between batches precisions at a HbA1c concentration of 8.0% was 5% and 7.5%, respectively. High density cholesterol (HDLc) was measured using a timed-endpoint direct homogenous assay on the same system, within and between batches precision (HDL 1.2 mmol/L) were 2.5% and 3%, respectively. Apolipoprotein A-1 (apoA1), apolipoprotein B (apoB) and Lp(a) were determined using rate nephelometric immunochemistry assay by IMMAGE system (Beckman, Richmond, California). [18] The within and between batches precision profile of Lp(a) at levels of 0.060 g/L, 0.40 g/l and 0.90 g/L were 3.2%, 2.7%, and 3.0%, and 3.5%, 3.1%, and 3.6%, respectively. Within and between batches precision profile for apoB (1.2 g/L) were 2.5% and 2.9%, respectively; for apoA-1 (1.05 g/L) were 3.4% and 3.9%, respectively. Low density cholesterol (LDLc) was calculated using the Friedwald formula and was not calculated when Tg level was >4.0 mmol/L.

### Statistical analysis

Data was analyzed with the Statistical Package for the Social Sciences Software (SPSS version 10). [19] Descriptive analysis including the estimation of mean values and standard error of the mean (SEM) for continuous variables were calculated Skewed parameters were logarithmically transformed and a parametric test was used. Correlations between the different lipid parameters and Lp(a) were carried out using Spearman correlation. Analysis of variance (ANOVA) was used to determine differences in subject characteristics. P value (two-tailed) < 0.05 was considered as statistically significant. A non-parametric test (Mann-Whitney) test was used to compare the correlation between lipid parameters and non-parametric variable like diabetes and IHD. Binary regression analysis was carried out to study the of lipid parameters on Lp(a) after adjusting for other observational variables like age, gender, BMI.

## Results

Lp(a), Tg, and HDLc were positively skewed, therefore, natural logarithms of the data were used in all parametric significance testing.

T2DM subjects had significantly lower concentration of Lp(a) (P = 0.012) and higher TG (P < 0.0001) concentration compared to non-diabetic subjects. Women had significantly higher Lp (a) and lower HDLc (P < 0.0001) concentration compared to men (Table 1).

**Table 1:** Shows the demographic, clinical and metabolic characteristics of T2DM compared to non-diabetic subjects.

	**Non-diabetic subjects**			**T2DM subjects**			
	**Females**	**Male**	**Both**	**Females**	**Males**	**Both**	**P-value (both)**
**Number**	69	87	156	86	135	221	
**Age (years)**	45(1.8)	43(1.7)	44(1.5)	47(0.9)	48(1.0)	47(0.8)	0.33
**BMI (kg/m^**2**^)**	33.4(2.6)	29.5(1.35)	30.7(1.26)	29.6(1)	30.5(0.8)	30.2(0.6)	0.71
**Tg (mmol/L)**	1.29 (1.12)	1.4(1.08)	1.36(1.06)	1.77(1.07)	1.86(1.06)	1.83(0.97)	<0.0001
**Tc (mmol/L)**	5.9 (0.28)	5.6(0.18)	5.7(0.15)	5.9(0.18)	5.8(0.14)	5.9(0.11)	0.59
**LDLc (mmol/L)**	3.9(0.26)	3.4(0.17)	3.7(0.13)	3.8(0.16)	3.7(0.11)	3.7(0.09)	0.97
**HDLc(mmol/L)**	1.37(1)	1.1(1)	1.18(1.02)	1.20(0.9)	1.1(0.91)	1.14(0.9)	0.21
**Lp(a) (g/L)**	0.246 (0.18–0.32)	0.15 (0.089–0.179)	0.18 (0.122–0.207)	0.165 (0.108–0.223)	0.101 (0.078–0.132)	0.123 (0.08–0.146)	0.015

Data are n, means +/- SEM

A significant Spearman correlation was revealed between Lp(a) and IHD among Omani T2DM subjects. The mean Lp(a) concentration was found to be higher among T2DM subjects with established IHD compared to T2DM without IHD. However, this did not reach statistical significance (P = 0.12) (Table 2). The proportions of T2DM subjects with IHD and an Lp(a) >0.3 g/L was higher compared to T2DM without IHD irrespective of gender, for women 42% vs. 27% and for men 17.5 vs. 8%, respectively.

**Table 2:** Shows the demographic, clinical and metabolic characteristics of T2DM subjects with established IHD compared those without IHD.

	**T2DM without-IHD**			**T2DM with IHD**			
	**Females**	**Males**	**Both**	**Females**	**Males**	**Both**	**P value Both**
**No**	66	83	149	20	52	72	
**Age**	44 (1.3)	41(1.2)	41.7 (1.14)	47 (2.8)	48 (1.4)	47.9 (1.3)	**0.001**
**BMI**	29.5(0.7)	32 (1.4)	31 (0.84	31.2 (0.95)	28 (0.60)	28.7 (0.55)	0.35
**TG (mmol/L)**	1.71(1.08))	2.1(1.08)	1.96(1.06)	1.91(1.16)	1.53(1.08)	1.61(1.07)	0.06
**Tc (mmol/L)**	6.0 (0.21)	5.8 (0.16)	5.9 (0.14	5.9 (0.35)	5.5 (0.18)	5.7 (0.16)	0.25
**LDLc(mmol/L)**	3.91 (0.19)	3.63 (0.17)	3.8 (0.13)	3.68 (0.43)	3.71 (0.14)	3.7 (0.15)	0.65
**HDL c (mmol/L)**	1.22 (1.03)	1.13 (1.03)	1.17 (1.02)	1.26 (1.06)	1.13 (1.03)	1.19 (1.03)	0.51
**Apo B (g/L)**	1.28 (0.05)	1.20 (0.04)	1.23 (0.03)	1.33 (0.09)	1.24 (0.04)	1.26 (0.04)	0.53
**ApoA1 (g/L)**	1.2 (0.04)	1.13 (0.02)	1.16 (0.02)	1.2 (0.08)	1.18 (0.03)	1.21 (0.03)	0.24
**Lp (a) g/L**	0.32 (0.021–1.14)	0.14 (0.09–0.42)	0.24 (0.021–1.14)	0.38 (0.11–1.73)	0.200 (0.07–0.78)	0.33 (0.090–1.73)	0.12

Data are n, means +/- SEM., for Lp(a) median and anti

Regression analysis adjusted for age, gender, BMI and HBA1_c_, revealed a significant (P = 0.002) negative correlation between Lp(a) and the triglycerides among T2DM subjects. In contrast, a significant positive correlation existed between Lp(a) and the apoB and LDLc (P = 0.001 and P = 0.02, respectively) among the non-diabetic subjects only (Table 3).

**Table 3:** Variables independently related to serum Lp(a) concentrations (log) in the Linear Regression Analysis.

	**B**	**SE of B**	**T**	**P value**
**Subjects with Type 2 diabetes**				
Log-TG	- 0.550	0.187	- 2.936	**0.002**
apo B	- 0.356	0.353	- 1.031	0.430
LDLc	0.098	0.080	1.231	0.221
Constant	- 1.884	0.532	- 3.542	0.001
				
**Non-diabetic subjects**				
Log-TG	- 0.0949	0.235	- 0.404	0.688
apo B	1.359	0.477	3.500	**0.001**
LDLc	0.256	0.100	2.534	**0.025**
Constant	- 2.964	0.636	- 6.618	<0.0001

B- slop, SE of B- standard error of the slop, T- T test. Variables also entered into the linear regression analysis but not included in the equation are, age, BMI, gender, HBA1c, HDL cholesterol.

## Discussion and conclusion

This study revealed that Lp(a) concentration was lower among Omani T2DM compared to non-diabetics subjects irrespective to gender. In contrast women had significantly higher Lp(a) concentration compared to men irrespective to the diabetic state. Similar findings to ours were also reported by Calmarza et al. and Gazzaruso et al., lower Lp(a) concentration noticed among T2DM compared to non-diabetics subjects [4,5]. In another study, Abdella et al., reported higher Lp(a) concentration among Kuwaiti T2DM women compared to men [20].

The present study revealed a significant negative correlation between Lp(a) and serum triglycerides among Omani T2DM subjects. This observation agrees with those reported by, Hernandex et al., using a different method for Lp(a) measurement [17]. In another study, lower concentrations of Lp(a) were reported in subjects with lipoprotein lipase deficiency [21], and it is tempting to speculate that diabetic subjects have some degree of functional deficiency of lipoprotein lipase that is implicated in the pathogenesis of hypertriglyceridaemia [22]. The likely explanation behind this inverse relationship is the fact that apo(a) is present in triglyceride-rich particles (TRPs). Apo(a)-containing TRPs, in parallel with chylomicron remnants, would be rapidly taken up by the liver through the remnant-receptor pathway. Thus, the lower levels of Lp(a) in patients with elevated triglyceride could be the result of the rapid catabolism of TRP apo(a) compared with the slower apo(a) catabolism in the LDL density range [17]. Supportive evidence to our findings was reported by Ko et al. Iin their study, improving insulin resistance in T2DM subjects by Rosiglitazone (Insulin sensitizing agent) was associated with lowering of triglycerides and increase in Lp(a) concentration [23].

The present study revealed that Lp(a) concentration is an independent risk factor for IHD among Omani T2DM subjects, irrespective of genders. Nevertheless, the actual concentration of Lp(a) had not reached statistical significance because of a number of limiting factors. Firstly; metabolic due to the accelerated catabolism of apo(a)-TRPs particles. Secondly, gender influence, having subdivided the studied groups by gender because of its confounding influence, the number of subjects within each subgroup became smaller and therefore, might have influenced the power of the study. A number of studies had also failed to show any significant association between Lp(a) and IHD among T2DM subjects without any emphasis on the reasons behind such findings [5,6,24]. On the contrary others have reported significant association between Lp(a) and IHD [9,25]. **In summary**. Lp(a) is an independent risk factor for IHD among Omani diabetic subjects. This present study highlights the complexity of lipoprotein metabolism and the impact of the different lipids parameters on Lp(a) concentration. Therefore, interpretation of Lp(a) should be cautiously exercised among diabetic subjects.

## Competing interests

The author(s) declare that they have no competing interests.

## Authors' contributions

AA is the major author of the above study, he had a substantial contribution to the conception, design, analysis as well as interpretation of the data. EM and LR have contributed to the drafting, revising as well as critically scrutinizing the manuscript. SA and MK have contributed to the recruitment and analysis of chemistry. AS contributed to the drafting, revising as well as critically scrutinizing the manuscript.

All authors have read and approved the manuscript.
